# Brain functional network topology and connectivity in primary blepharospasm

**DOI:** 10.3389/fnsys.2025.1654795

**Published:** 2025-10-08

**Authors:** Xin-Xin Yao, Hua-Liang Li, Shu Wang, Si-Yu Gu, Jian-Bin Hu, Wen-Hui Li, Ping-Lei Pan

**Affiliations:** ^1^Department of Radiology, Affiliated Hospital 6 of Nantong University, Yancheng Third People’s Hospital, Yancheng, China; ^2^Department of Neurology, Affiliated Hospital 6 of Nantong University, Yancheng Third People’s Hospital, Yancheng, China

**Keywords:** blepharospasm, resting-state fMRI, graph theory, network topology, functional connectivity

## Abstract

**Background:**

The pathophysiology of primary blepharospasm (BSP) remains incompletely understood. This study aimed to characterize whole-brain functional network topology in treatment-naive BSP patients.

**Methods:**

Thirty-nine treatment-naive BSP patients and 39 matched healthy controls (HCs) underwent resting-state fMRI. Graph theoretical analysis was applied to assess global and nodal network metrics. Network-Based Statistics (NBS) identified subnetworks with altered functional connectivity (FC). Correlations between network metrics and clinical variables [Jankovic Rating Scale (JRS), illness duration] were explored.

**Results:**

Compared to HCs, BSP patients exhibited significantly lower local efficiency [*p* = 0.0002, false discovery rate (FDR) corrected], while global efficiency, characteristic path length, clustering coefficient, normalized clustering coefficient, normalized characteristic path length, or small-worldness were preserved (all *p* > 0.05, FDR corrected). Nodal analysis revealed decreased efficiency/degree in the bilateral thalamus and left supplementary motor area, and increased efficiency/degree in the bilateral precentral gyri, right postcentral gyrus, and left insula (all *p* < 0.05, FDR corrected). NBS identified subnetworks with altered FC across sensorimotor, limbic-subcortical, frontoparietal, and default mode networks, featuring both hyper- and hypo-connectivity (*p* < 0.05, NBS-corrected). Notably, left thalamic efficiency negatively correlated with illness duration (*r* = −0.481, *p* = 0.0019), and right precentral gyrus efficiency positively correlated with JRS total score (*r* = 0.395, *p* = 0.0129).

**Conclusion:**

BSP is characterized by complex functional network disruptions, including impaired local information processing, altered nodal importance in key motor and relay hubs, and widespread connectivity changes. These findings reinforce BSP as a network disorder. These network alterations may serve as objective markers for disease progression and could guide the development of targeted neuromodulation therapies.

## Introduction

Primary blepharospasm (BSP) is a debilitating adult-onset focal dystonia characterized by involuntary, repetitive, bilateral contractions of the orbicularis oculi muscles, often leading to spasmodic eyelid closure (Defazio et al., 2017). While considered relatively uncommon, epidemiological studies suggest prevalence rates varying across populations, affecting about 16–133 cases per million, with a peak onset typically occurring between the fifth and seventh decades of life and a marked predominance in females (Valls-Sole and Defazio, 2016; Sun et al., 2018). This disease imposes a substantial burden on patients, frequently causing functional blindness despite intact vision, which severely impairs essential daily activities such as reading, driving, and navigating public spaces, leading to social isolation and reduced independence (Sun et al., 2018). Beyond the motor symptoms, BSP is increasingly recognized for its significant non-motor manifestations, including anxiety, depression, sleep disturbances, photosensitivity, and pain, which further diminish quality of life and contribute to the overall disease burden (Ferrazzano et al., 2019; Digre, 2015; Zhou et al., 2023). Current therapeutic strategies, primarily revolving around repeated periocular injections of botulinum neurotoxin (BoNT), offer effective symptomatic relief for many but face limitations such as temporary efficacy requiring lifelong treatment cycles, potential side effects like ptosis, variable treatment response, and a failure to address the underlying disease process or associated non-motor complaints (Simpson et al., 2016; Mitsikostas et al., 2021; Fang et al., 2020; Duarte et al., 2020). Despite extensive investigation yielding valuable insights, the precise pathophysiological mechanisms driving primary BSP remain incompletely understood, although converging evidence points towards dysfunction within a complex cortico-striato-thalamo-cerebellar brain network, involving aberrant sensorimotor integration and maladaptive neuroplasticity (Defazio et al., 2017; Valls-Sole and Defazio, 2016; Zhu et al., 2023; Peterson and Sejnowski, 2017; Fagan et al., 2021; Evinger, 2015).

Resting-state functional magnetic resonance imaging (rs-fMRI), without requiring active task performance, has emerged as a powerful non-invasive tool to investigate the brain’s intrinsic functional architecture and its alterations in neurological disorders, including dystonia (Barkhof et al., 2014; Smith et al., 2013). Previous rs-fMRI studies in BSP have revealed abnormalities in both local brain activity (e.g., altered amplitude of low-frequency fluctuations or regional homogeneity) and functional connectivity (FC) within and between key networks. Notably, disruptions have been reported involving the basal ganglia-thalamo-cortical circuits, sensorimotor network (SMN), visual network, default mode network (DMN), and cerebellum (Jiang et al., 2019; Pan et al., 2021; Liu et al., 2023; Ni et al., 2017; Fang et al., 2021; Jochim et al., 2018; Luo et al., 2022). While informative, these studies often focus on regional activity or pairwise connectivity. Graph theory analysis offers a more comprehensive systems-level approach, allowing us to characterize the topological properties of the entire brain network, such as its efficiency of information transfer, balance between integration and segregation, and the centrality of specific brain regions (Rubinov and Sporns, 2010; Sporns, 2018). Applying this framework is crucial for understanding BSP as a potential brain network disorder (Cheng et al., 2023; Hou et al., 2022). However, a significant limitation of many prior neuroimaging studies is the inclusion of patients already receiving BoNT treatment. Growing evidence indicates that BoNT therapy itself induces structural and functional neuroplastic changes within the central nervous system (Alexandru et al., 2016; Jochim et al., 2018; Feng et al., 2021; Nevrlý et al., 2018; Hok et al., 2021), potentially confounding the interpretation of disease-inherent pathophysiology. Therefore, investigating the brain functional network topology using graph theory in treatment-naive BSP patients offers a distinct advantage. This approach allows for the characterization of intrinsic network alterations specific to the disease state, free from treatment-induced effects, thereby providing a clearer understanding of the fundamental pathophysiological mechanisms underlying primary blepharospasm.

Therefore, the primary objective of this study was to utilize graph theoretical analysis of rs-fMRI data to characterize the topological organization of whole-brain functional networks (Wang et al., 2015) in treatment-naive BSP patients. To identify specific subnetworks exhibiting altered FC strength in BSP patients, the network-based statistics (NBS) analysis was additionally conducted (Zalesky et al., 2010). Furthermore, we investigated whether specific alterations in network topology/connectivity correlated with clinical variables, such as illness severity and duration. Grounded in established evidence of sensorimotor integration deficits and cortico-basal ganglia-thalamocortical (CBGTC) circuit dysfunction in dystonia, we hypothesized that treatment-naive BSP patients, relative to healthy controls, would demonstrate: (1) disrupted local information processing, reflected by reduced local efficiency; (2) altered nodal centrality in key sensorimotor and subcortical hubs; and (3) abnormal FC within the CBGTC network.

## Materials and methods

### Study participants

Between October 2023 and October 2024, we recruited 39 treatment-naive patients with BSP (Jankovic et al., 2009) and 39 healthy controls (HCs) matched for sex, age, and education level at the Sixth Affiliated Hospital of Nantong University. Inclusion criteria for BSP patients were: (1) meeting clinical diagnostic criteria for primary BSP; (2) having no contraindications to MRI and the ability to tolerate the scanning procedure with good cooperation; (3) being treatment-naive, with no prior history of BoNT injections or systemic medications specifically for BSP. Exclusion criteria for all participants included: history or current diagnosis of other neurological disorders, major psychiatric illness (past or present), significant ophthalmological conditions that could potentially confound symptoms, history of alcohol or drug abuse, a family history of movement disorders, and the presence of structural brain lesions identified on conventional MRI sequences. HCs were required to be in good general health with no history of neurological or psychiatric disorders. The study protocol adhered to the principles of the Declaration of Helsinki and was approved by the Ethics Committee of the Affiliated Hospital 6 of Nantong University (approval no: 201823). Written informed consent was obtained from all participants prior to enrollment.

### Clinical assessment

All participants underwent demographic assessment including age, sex, and years of education. For BSP patients, a detailed medical history including disease duration was obtained. Symptom severity was evaluated using the Jankovic Rating Scale (JRS) (Jankovic et al., 2009). Neuropsychological status was assessed using the Self-Rating Anxiety Scale (SAS) and the Self-Rating Depression Scale (SDS) to evaluate anxiety and depression levels, respectively (Zung, 1971; Zung, 1965). The diagnosis of BSP was confirmed by an experienced neurologist (H.L.L.). Following clinical assessments, all participants underwent brain rs-fMRI scanning.

### Magnetic resonance image acquisition

MR scans were performed on a GE Discovery 750 W 3.0 T scanner (GE Healthcare, Milwaukee, WI, United States) equipped with an 8-channel dedicated head coil. During scanning, participants were instructed to remain awake, keep their eyes closed, lie still, and minimize directed thought. Foam padding and earplugs were used to reduce head motion and scanner noise. Resting-state fMRI data were acquired using a gradient-recalled echo-planar imaging (GRE-EPI) sequence with the following parameters: Repetition Time (TR) = 2000 ms; Echo Time (TE) = 30 ms; Flip Angle (FA) = 90°; Field of View (FOV) = 240 × 240 mm^2^; Matrix size = 64 × 64; Slice number = 35; Slice thickness = 4 mm; No inter-slice gap; Number of excitations (NEX) = 1; Voxel size = 3.75 × 3.75 × 4 mm^3^. A total of 230 volumes were acquired. The 35 contiguous axial slices covered the entire brain and were acquired parallel to the anterior commissure-posterior commissure (AC-PC) line.

### Rs-fMRI data processing

#### Preprocessing

Rs-fMRI data were preprocessed using the Data Processing & Analysis for Brain Imaging (DPABI) toolbox (v8.2) (Yan et al., 2016), which is based on MATLAB (The MathWorks Inc., Natick, MA, United States), utilizing the Data Processing Assistant for Resting-State fMRI (DPARSF) module (Chao-Gan and Yu-Feng, 2010). The preprocessing pipeline included the following steps: (1) conversion of DICOM images to NIFTI format; (2) removal of the first 10 volumes to allow for MR signal stabilization; (3) slice-timing correction; (4) head motion correction (participants with head motion exceeding 2.0 mm of translation or 2.0° of rotation in any direction were excluded from further analysis); (5) spatial normalization to Montreal Neurological Institute (MNI) space using EPI templates and resampling to 3 × 3 × 3 mm^3^ voxels; (6) spatial smoothing with a 6 mm Full Width at Half Maximum (FWHM) Gaussian kernel; (7) removal of linear trends; (8) temporal band-pass filtering (0.01–0.1 Hz); (9) nuisance covariate regression, removing signals from white matter and cerebrospinal fluid (mean signals), as well as 24 head motion parameters derived from the Friston 24-parameter model. We did not perform global signal regression to avoid introducing spurious negative correlations. Frame-wise displacement (FD) was calculated, and scrubbing was performed based on FD > 0.5 mm.

#### Functional network construction

Whole-brain functional networks were constructed for each participant. Network nodes were defined using the Automated Anatomical Labeling (AAL) atlas, partitioning the brain into 116 regions of interest (ROIs). The mean time series was extracted for each ROI from the preprocessed fMRI data. Network edges were defined by calculating the Pearson correlation coefficient between the mean time series of all pairs of ROIs, resulting in a 116 × 116 FC matrix for each participant. These correlation coefficients were Fisher *r*-to-*z* transformed to improve normality before further analysis. To construct unweighted binary networks for graph theoretical analysis, the individual Fisher *r*-to-*z* transformed correlation matrices were converted into binary matrices using a range of predefined absolute correlation thresholds (S) from 0.05 to 0.40, with an increment of 0.01. This range was selected to ensure that the resulting networks were not fragmented at lower thresholds or overly dense at higher thresholds, and it is consistent with approaches used in prior graph theoretical studies of brain networks to ensure that results are not dependent on an arbitrary single threshold (Suo et al., 2022). For each threshold S, if the absolute correlation coefficient between two ROIs was greater than S, the corresponding element in the binary matrix was set to 1 (indicating an edge); otherwise, it was set to 0.

#### Graph theoretical analysis of brain functional networks

Graph theoretical analysis was performed using the Graph Theoretical Network Analysis (GRETNA) toolbox (Wang et al., 2015) to quantify the topological properties of the constructed binary brain networks. For each participant and each threshold S within the defined range, global and nodal network metrics were calculated. To provide summary measures independent of a single arbitrary threshold, the area under the curve (AUC) was computed for each metric across the entire range of thresholds. Global metrics included: clustering coefficient (C_p_), characteristic path length (L_p_), normalized clustering coefficient (*γ*), normalized characteristic path length (*λ*), small-worldness (*σ*), global efficiency (E_g_), and local efficiency (E_loc_). Nodal metrics included: nodal degree, nodal efficiency, and nodal betweenness centrality.

#### Network-based statistics (NBS) analysis

To identify specific subnetworks exhibiting altered FC strength between BSP patients and HCs, the NBS approach was employed (Zalesky et al., 2010). NBS assesses differences in FC strength across all pairwise connections while controlling for the family-wise error rate (FWER). The procedure involved: (1) Calculating an independent two-sample *t*-test for each connection (edge) in the Fisher *r*-to-*z* transformed FC matrices to compare connectivity strength between the BSP and HC groups, controlling for age, sex, education, SAS, and SDS scores via a general linear model framework. (2) Applying a primary component-forming threshold (*t*-value corresponding to *p* < 0.001, uncorrected) to the resulting *t*-statistic map to identify a set of supra-threshold connections. (3) Identifying connected components (subnetworks) formed by these supra-threshold connections. (4) Assessing the statistical significance of each identified component using a non-parametric permutation test (5,000 permutations). In each permutation, participant group labels were randomly shuffled, the same *t*-tests were performed, and the maximal component size (number of connections) was recorded to build a null distribution of maximal component sizes. (5) Components in the original data were considered statistically significant if their size exceeded the 95th percentile of the maximal component size distribution derived from the permutations, corresponding to a component-level significance threshold of *p* < 0.05 (FWER-corrected). This method identified specific interconnected subnetworks where FC significantly differed between groups.

#### Statistical analysis

Statistical analyses for demographic, clinical, and graph theoretical metrics were performed using SPSS version 27.0 (IBM Corp., Armonk, NY, United States). Normality of demographic and clinical data was assessed using the Shapiro–Wilk test. Group differences in normally distributed continuous variables (age, education level, illness duration, SAS score, SDS score) were assessed using independent two-sample *t*-tests, with results reported as mean ± standard deviation (SD). Non-normally distributed variables (JRS score) were compared between groups using the Mann–Whitney U test, with results reported as median (interquartile range). The distribution of sex between the two groups was compared using the Chi-square (χ^2^) test. A significance level of *p* < 0.05 was used for these demographic and clinical comparisons.

Group differences between patients with BSP and HCs in the global network topological metrics (C_p_, L_p_, *γ*, *λ*, *σ*, E_g_, E_loc_) and nodal topological metrics (nodal degree, nodal efficiency, and nodal betweenness centrality) were assessed using two-sample *t*-tests, controlling for age, sex, education level, SAS score, SDS score, and FD as covariates. The statistical significance threshold for global metrics was set at *p* < 0.05, false discovery rate (FDR) correction.

Within the BSP patient group, partial correlation analyses were conducted to explore the relationship between the AUC values of global/nodal topological metrics and the mean connectivity strength within NBS subnetworks that showed significant group differences and clinical variables (JRS score, disease duration). These correlations were controlled for age, sex, education level, SAS score, SDS score, and FD. Statistical significance for the correlation analyses was set at *p* < 0.05.

## Results

### Demographic and clinical characteristics

A total of 39 treatment-naive BSP patients (14 males, 25 females; mean age 51.10 ± 6.58 years) and 39 HCs (14 males, 25 females; mean age 50.13 ± 5.35 years) were included. As shown in Table 1, there were no significant differences in age, sex, or education level between patients with BSP and HCs (all *p* > 0.05). However, the BSP group exhibited significantly higher scores on the SAS (42.46 ± 7.30 vs. 36.31 ± 5.27, *t* = 4.27, *p* < 0.001, Cohen’s *d* = 0.97) and SDS (44.21 ± 7.21 vs. 38.21 ± 5.21, *t* = 4.21, *p* < 0.001, Cohen’s *d* = 0.95) compared to the HC group.

**Table 1:** Demographic and clinical characteristics of all subjects.

Characteristics	Primary blepharospasm	Healthy controls	*t/χ2*	*p*
Gender, M/F	14/25	14/25	0	1^a^
Age, y	51.10 ± 6.58	50.13 ± 5.35	0.72	0.48^b^
Education, y	8.90 ± 3.36	9.51 ± 3.88	−0.75	0.46^b^
Illness duration, m	12.62 ± 4.92	–	–	–
JRS total score	3 (2, 5)^c^	–	–	–
JRS severity subscale	1 (1, 2)^c^	–	–	–
JRS frequency subscale	2 (1, 3)^c^	–	–	–
SAS	42.46 ± 7.30	36.31 ± 5.27	4.27	< 0.001^b^
SDS	44.21 ± 7.21	38.21 ± 5.21	4.21	< 0.001^b^

a The *p* value for gender distribution was obtained by chi-square test.

b The *p* values were obtained by two-sample t-tests.

c Data are represented as median (quartiles). Data was presented as mean ± standard deviation unless otherwise specified. M, male; F, female; y, years; m, months; JRS, Jankovic Rating Scale; SAS, self-rating anxiety scale; SDS, self-rating depression scale.

### Group differences in global metrics

**Figure 1:**
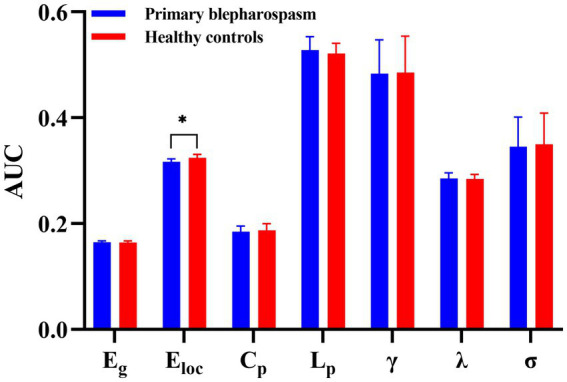
Group differences in global metrics. No significant group differences between patients with primary blepharospasm and healthy controls were found in global efficiency (E_g_), characteristic path length (L_p_), clustering coefficient (C_p_), normalized clustering coefficient (*γ*), normalized characteristic path length (*λ*), or small-worldness (*σ*) (all *p* > 0.05, false discovery rate corrected). However, local efficiency (E_loc_) was significantly lower in the primary blepharospasm group compared to healthy controls (^*^p < 0.05 after false discovery rate correction). AUC, area under the curve.

No significant group differences were found in global efficiency (E_g_), characteristic path length (L_p_), clustering coefficient (C_p_), normalized clustering coefficient (*γ*), normalized characteristic path length (*λ*), or small-worldness (*σ*) (all *p* > 0.05, FDR corrected). However, local efficiency (E_loc_) was significantly lower in the BSP group compared to HCs (0.3169 ± 0.0054 vs. 0.3243 ± 0.0067, *p* = 0.0002, Cohen’s *d* = −1.22, FDR corrected). These findings are summarized in Table 2 and Figure 1.

**Table 2:** Between-group differences in the AUC values of global metrics.

Metrics	Primary blepharospasm (*n* = 39)	Healthy controls (*n* = 39)	*p*
E_g_	0.1646 (0.0026)	0.1642 (0.0029)	0.3164
E_loc_	**0.3169 (0.0054)**	**0.3243 (0.0067)**	**0.0002**
C_p_	0.1850 (0.0103)	0.1871 (0.0127)	0.9245
L_p_	0.5279 (0.0254)	0.5211 (0.0199)	0.8003
γ	0.4832 (0.0641)	0.4855 (0.0689)	0.5415
λ	0.2855 (0.0103)	0.2843 (0.0084)	0.3798
σ	0.3455 (0.0557)	0.3499 (0.0589)	0.7147

All data are expressed as mean (standard deviation). *p* < 0.05 after false discovery rate correction. AUC, area under the curve. E_g_, global efficiency; E_loc_, local efficiency; C_p_, clustering coefficient; L_p_, characteristic path length; γ, normalized clustering coefficient; λ, normalized characteristic path length; σ, small worldness. Bold values indicate statistical significance.

### Group differences in nodal metrics

**Figure 2:**
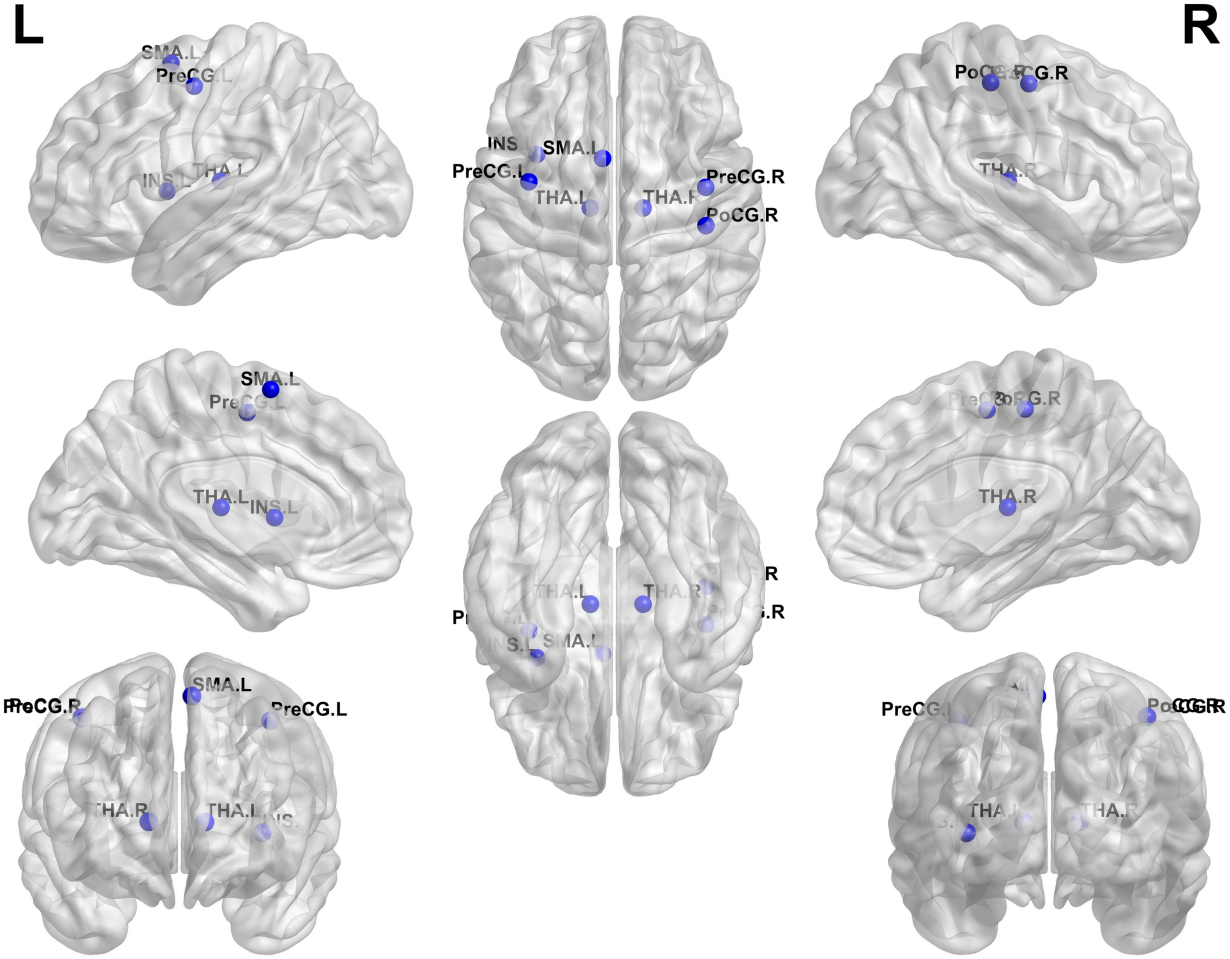
Group differences in nodal metrics. Compared to healthy controls, patients with primary blepharospasm showed altered nodal degree and/or nodal efficiency/nodal betweenness in several brain regions, including the left thalamus (THA. L), right thalamus (THA. R), left supplementary motor area (SMA. L), left precentral gyrus (PreCG. L), right precentral gyrus (PreCG. R), right postcentral gyrus (PoCG. R), and left insula (INS. L) (all *p <* 0.05, false discovery rate corrected). L, left; R, right.

Compared to HCs, patients with BSP showed significantly decreased nodal degree and/or nodal efficiency in several brain regions, including the left thalamus, right thalamus, and left supplementary motor area (all *p <* 0.05, FDR corrected). Conversely, BSP patients exhibited significantly increased nodal degree and/or nodal efficiency in the bilateral precentral gyrus, right postcentral gyrus, and left insula (all *p <* 0.05, FDR corrected). Detailed results are presented in Table 3 and Figure 2.

**Table 3:** Differences in nodal metrics between patients with primary blepharospasm group and healthy controls.

Group and brain region	*p*		
	Nodal degree	Nodal efficiency	Nodal betweenness
Primary blepharospasm < healthy controls			
Left thalamus	**0.0084**	**0.0032**	0.0566
Right thalamus	**0.0118**	**0.0068**	0.0508
Left supplementary motor area	0.0592	**0.0146**	0.1325
Primary blepharospasm > healthy controls			
Right precentral gyrus	**0.0026**	**0.0012**	**0.0294**
Left precentral gyrus	**0.0068**	**0.0024**	0.0562
Left insula	0.0529	**0.0099**	0.2683
Right postcentral gyrus	**0.0327**	**0.0062**	0.3521

Regions were deemed significant for between-group differences in at least one nodal centrality measures (*p* < 0.05, false discovery rate corrected indicated in bold).

### Group differences in functional network connectivity

**Figure 3:**
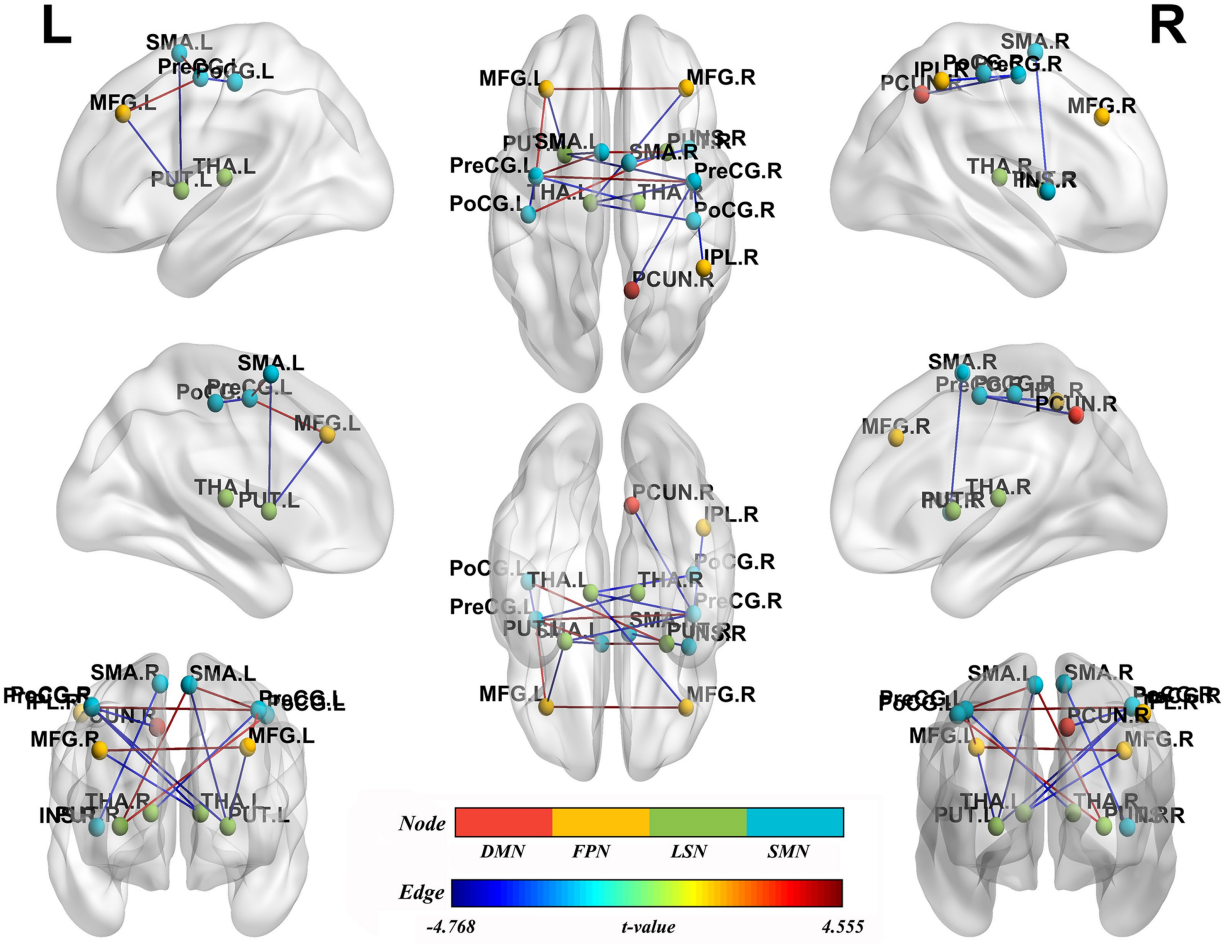
Group differences in functional network connectivity. Nodes represent specific brain regions grouped by functional networks, including the default mode network (DMN), fronto-parietal network (FPN), limbic-subcortical network (LSN), and sensorimotor network (SMN). Edges indicate significant changes in functional connectivity between patients with primary blepharospasm and healthy controls, with edge colors reflecting the direction and magnitude of *t*-values (all *p* < 0.05, Network-Based Statistic corrected). L, left; R, right; PUT.L, left putamen; LSN, limbic-subcortical network; PreCG.R, right precentral gyrus; SMN, sensorimotor network; SMA.L, left supplementary motor area; MFG.L, left middle frontal gyrus; FPN, fronto-parietal network; PUT.R, right putamen; PoCG.L, left postcentral gyrus; THA.L, left thalamus; PoCG.R, right postcentral gyrus; THA.R, right thalamus; PreCG.L, left precentral gyrus; MFG.R, right middle frontal gyrus; IPL.R, right inferior parietal lobule; SMA.R, right supplementary motor area; INS.R, right insula; PCUN.R, right precuneus; DMN, default mode network.

NBS analysis identified significant brain network components exhibiting altered FC between BSP patients and HCs (*p* < 0.05, NBS corrected). The networks primarily involved regions within and between the SMN, limbic-subcortical network (LSN), frontoparietal network (FPN), and DMN. Specifically, compared to HCs (Table 4; Figure 3), BSP patients showed significantly increased FC between: the left precentral gyrus and the right precentral gyrus (*t* = 4.555), the left supplementary motor area and the left precentral gyrus (*t* = 4.363), the left supplementary motor area and the right putamen (*t* = 4.094), the right putamen and the left postcentral gyrus (*t* = 3.875), and the left middle frontal gyrus and the left precentral gyrus (*t* = 3.768). Conversely, BSP patients demonstrated significantly decreased FC between: the left putamen and the right precentral gyrus (*t* = −4.589), the left putamen and the left supplementary motor area (*t* = −4.768), the left putamen and the left middle frontal gyrus (*t* = −4.254), the left thalamus and the right postcentral gyrus (*t* = −3.784), the left thalamus and the right precentral gyrus (*t* = −4.137), the left thalamus and the right middle frontal gyrus (*t* = −3.768), the right thalamus and the left precentral gyrus (*t* = −3.589), the left postcentral gyrus and the left precentral gyrus (*t* = −3.902), the right inferior parietal lobule and the right precentral gyrus (*t* = −3.632), the right supplementary motor area and the right insula (*t* = −3.589), and the right precuneus and the right precentral gyrus (*t* = −4.225).

**Table 4:** Altered functional network connectivity in primary blepharospasm.

Brain region	Corresponding network	Brain region	Corresponding network	*p*	*t*
PUT.L	LSN	PreCG.R	SMN	0.0004	−4.589
PUT.L	LSN	SMA.L	SMN	0.0002	−4.768
PUT.L	LSN	MFG.L	FPN	0.0006	−4.254
PUT.R	LSN	PoCG.L	SMN	0.0001	3.875
THA.L	LSN	PoCG.R	SMN	0.0003	−3.784
THA.L	LSN	PreCG.R	SMN	0.0005	−4.137
THA.R	LSN	PreCG.L	SMN	0.0003	−3.589
THA.L	LSN	MFG.R	FPN	0.0004	−3.768
PoCG.L	SMN	PreCG.L	SMN	0.0001	−3.902
IPL.R	FPN	PreCG.R	SMN	0.0007	−3.632
SMA.L	SMN	PreCG.L	SMN	0.0006	4.363
SMA.L	SMN	PUT.R	LSN	0.0006	4.094
SMA.R	SMN	INS.R	SMN	0.0008	−3.589
MFG.L	FPN	PreCG.L	SMN	0.0008	3.768
PCUN.R	DMN	PreCG.R	SMN	0.0006	−4.225
PreCG.L	SMN	PreCG.R	SMN	0.0001	4.555

Brain network component exhibited significantly altered functional connectivity in patients with primary blepharospasm compared to healthy controls, as identified using the Network-Based Statistic (NBS) method (*p* < 0.05, NBS corrected). PUT.L, left putamen; LSN, limbic-subcortical network; PreCG.R, right precentral gyrus; SMN, sensorimotor network; SMA.L, left supplementary motor area; MFG.L, left middle frontal gyrus; FPN, fronto-parietal network; PUT.R, right putamen; PoCG.L, left postcentral gyrus; THA.L, left thalamus; PoCG.R, right postcentral gyrus; THA.R, right thalamus; PreCG.L, left precentral gyrus; MFG.R, right middle frontal gyrus; IPL.R, right inferior parietal lobule; SMA.R, right supplementary motor area; INS.R, right insula; PCUN.R, right precuneus; DMN, default mode network.

### Correlations between nodal metrics and clinical features

**Figure 4:**
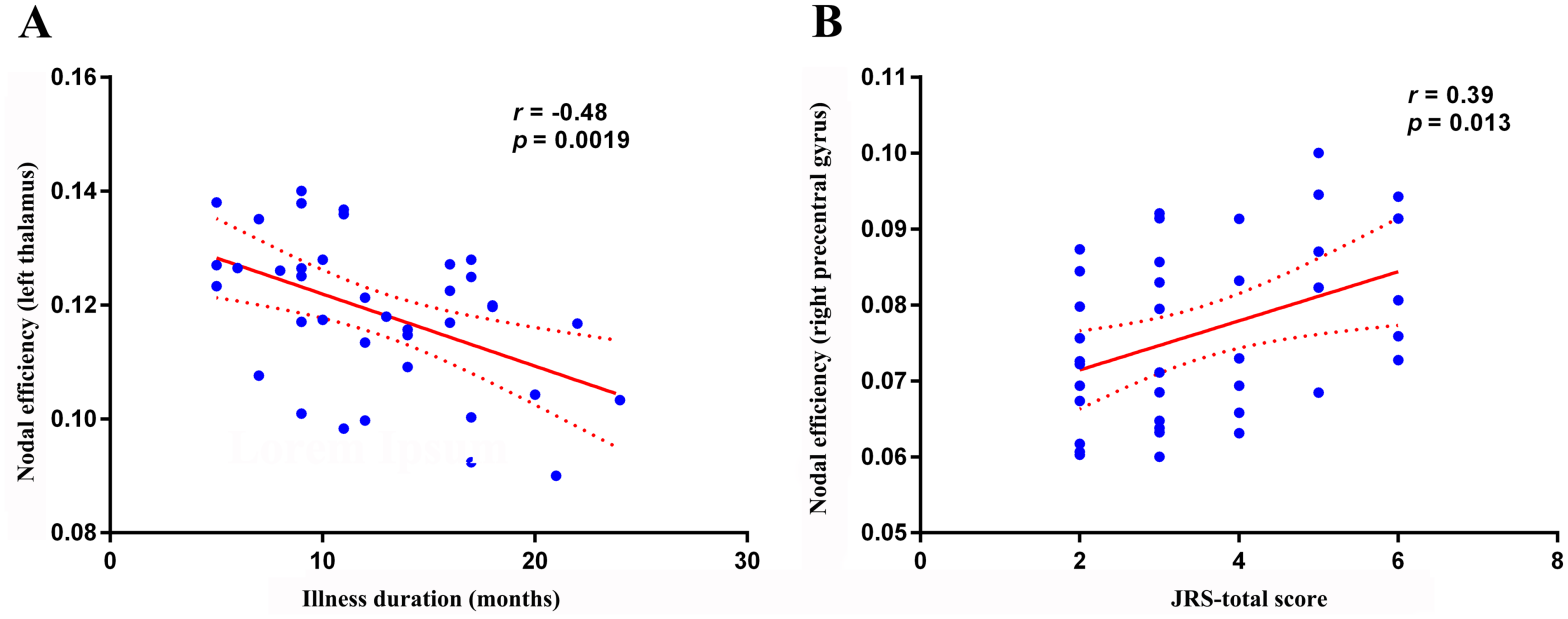
Correlation analysis. **(A)** Within the primary blepharospasm group, nodal efficiency of the left thalamus was negatively correlated with disease duration (*r* = −0.481, *p* = 0.0019). **(B)** nodal efficiency of the right precentral gyrus was positively correlated with the JRS total score (*r* = 0.395, *p* = 0.0129). JRS, Jankovic Rating Scale.

Within the BSP group, nodal efficiency of the left thalamus was negatively correlated with illness duration (Figure 4A, *R* = − 0.481, *p* = 0.0019). Furthermore, nodal efficiency of the right precentral gyrus was positively correlated with the JRS total score (Figure 4B, *R* = 0.395, *p* = 0.0129). No significant correlations were observed between any of the global topological metrics, the mean connectivity strength of the NBS-identified subnetwork, or the other nodal metrics with any of the assessed clinical variables (JRS score, disease duration) after correction for multiple comparisons (all *p* > 0.05).

## Discussion

This study employed graph theoretical analysis of rs-fMRI data to comprehensively investigate the whole-brain functional network topology in a cohort of untreated patients with BSP. Our findings reveal a complex landscape of network alterations, characterized by: (1) globally reduced efficiency of local information processing (decreased E*_loc_*) while maintaining overall network integration (preserved E_g_, L_p_, C_p_, *γ*, and *λ*) and small-world architecture; (2) bidirectional alterations in nodal importance, with decreased efficiency/centrality in core relay and planning hubs like the thalamus and supplementary motor area, alongside increased efficiency/centrality in sensorimotor regions (precentral gyrus, postcentral gyrus) and the insula; (3) clinically relevant correlations, linking reduced left thalamic efficiency to longer disease duration and increased right precentral gyrus efficiency to greater disease severity (JRS score); and (4) distributed subnetworks identified via NBS showing altered connectivity strength, encompassing nodes within SMN, LSN, FPN, and DMN, featuring both pathologically enhanced and reduced connections. These results solidify the understanding of BSP as a complex network disorder and provide valuable insights into its pathophysiology, aligning with and extending findings across the broader dystonia spectrum.

### Impaired local processing efficiency and core hub dysfunction

The significant reduction in global local efficiency (E_loc_) in our untreated BSP cohort suggests impaired functional segregation and less efficient information transfer within localized functional modules (Rubinov and Sporns, 2010). This finding resonates with reports of disrupted topological organization or reduced local efficiency in other cohorts of blepharospasm (Hou et al., 2022) and certain dystonia subtypes (Li et al., 2020; Jin et al., 2011; Norris et al., 2020), potentially reflecting a common vulnerability in network modularity. While global efficiency and small-worldness were preserved, consistent with findings in some other focal dystonia cohorts (Hou et al., 2022; Giannì et al., 2022), suggesting the overall network architecture remains relatively intact for global integration, the specific deficit in local processing might be a sensitive early indicator of dysfunction, hindering the finely tuned, coordinated activity necessary for normal sensorimotor control, particularly involving the facial region where abnormalities are consistently reported in BSP.

At the nodal level, our finding of reduced node efficiency and/or degree centrality in the thalamus and SMA in patients with blepharospasm points to dysfunction within critical nodes of the classical motor control loops. This observation aligns with previously reported alterations in brain structure, regional function, or FC across various focal and generalized dystonias, identified using diverse neuroimaging techniques, collectively suggesting a wider network dysregulation involving the CBGTC and cerebello-thalamo-cortical circuits (Zhu et al., 2023; Giannì et al., 2022; Huang et al., 2022a; Zheng et al., 2012; Løkkegaard et al., 2015; Hendrix and Vitek, 2012; Mascia et al., 2020). The thalamus, a key relay station and gatekeeper (Giannì et al., 2022; Hendrix and Vitek, 2012; Moustafa et al., 2017), exhibits changes in multiple dystonias, although the direction and pattern of these alterations are inconsistent (Giannì et al., 2022; Huang et al., 2022a; Zheng et al., 2012; Løkkegaard et al., 2015; Zhang et al., 2022). Specifically, for BSP, Zhang et al. (2022) conducted a multimodal meta-analysis of whole-brain voxel-based morphometry (VBM) studies and functional imaging studies that identified hyperactivity in the left thalamus. Our observation of reduced nodal efficiency/centrality, despite potential hyperactivity reported elsewhere, could reflect a dissociation between local processing levels and the node’s overall effectiveness in network integration. Regardless of the specific pattern, such disruption could impair the integration and relay of sensory information crucial for eyelid control (Scorr et al., 2022). The negative correlation we observed between left thalamic efficiency and disease duration further suggests a progressive functional decline in its network role. Similarly, the SMA, vital for motor planning and sequencing, frequently shows abnormalities in BSP and other dystonias, again with heterogeneous patterns (Liu et al., 2023; Giannì et al., 2022; Huang et al., 2022a; Løkkegaard et al., 2015; Zhang et al., 2022; Xu et al., 2023; Xu et al., 2024; Kshatriya et al., 2024; Huang et al., 2022b). A meta-analysis of VBM studies showed increased gray matter in the SMA, while the meta-analysis of functional studies revealed hypoactivity in the SMA in BSP (Zhang et al., 2022). Similar findings also existed in cervical dystonia (Huang et al., 2022a). However, gray matter reduction (Huang et al., 2022b) and task-related activation decrease (Løkkegaard et al., 2015) in the SMA was also found in idiopathic dystonia. Notably, Xu et al. using VBM and causal structural covariance network analysis, found increased bilateral SMA gray matter volume and increased grey matter volume covariance between the right SMA and right brainstem, left superior frontal gyrus, left SMA and left paracentral gyrus in blepharospasm (Xu et al., 2023). Further causal structural covariance network, modularity analysis and functional decoding showed that the right SMA served as a driving core in patients with blepharospasm. This highlights a potentially early and critical role for the SMA in BSP (Xu et al., 2023). The functional dysregulation of these core thalamic and SMA hubs, despite manifesting heterogeneously across studies, consistently points towards functional disruption within critical nodes of the classical motor control and sensory integration networks. This disruption likely represents a fundamental breakdown or dysregulation in the network’s ability to integrate sensory information and prepare appropriate motor commands and may contribute to the observed symptoms of BSP.

### Sensorimotor and insular hyper-centrality: maladaptation or compensation?

Our finding of increased nodal efficiency/centrality in the bilateral precentral gyrus (M1), right postcentral gyrus (S1), and left insula suggests these regions assume heightened importance within the blepharospasm network. The sensorimotor cortex (SMC = M1 + S1) is central to sensorimotor integration, a process known to be deficient in dystonia (Zhu et al., 2023; Quartarone and Hallett, 2013). This hyper-centrality raises a critical question: does it represent a primary maladaptive process driving symptoms, or is it a compensatory mechanism attempting to overcome underlying dysfunction? Evidence from neuroimaging studies across different focal dystonias reveals a complex, often dissociated, pattern of structural and functional changes in these sensorimotor and insular regions (Huang et al., 2022a; Zhang et al., 2022; Huang et al., 2022b). Structurally, our finding of heightened network centrality aligns with meta-analyses reporting consistently increased gray matter volume (GMV) in the bilateral precentral and postcentral gyri and right insula across various idiopathic dystonias (Huang et al., 2022b) and specifically in blepharospasm (Zhang et al., 2022). This structural hypertrophy could, in part, underpin the increased network importance observed functionally in our study.

However, the functional picture is more nuanced. Multimodal meta-analyses often report functional hypoactivity in these same regions despite increased GMV. For instance, in blepharospasm, Zhang et al. (2022) found increased GMV in precentral/postcentral gyri but functional hypoactivity in the left precentral gyrus and left insula, with conjoint analysis showing increased GMV accompanied by hypoactivity in the left precentral gyrus. Similarly, in cervical dystonia, increased GMV in the right precentral/postcentral gyri overlapped with functional hypoactivity (Huang et al., 2022a). An earlier functional meta-analysis across dystonias also found decreased task-related activation in the left postcentral gyrus (hand area) and left SMA, although task-specific increases were seen in other S1/S2 areas (Løkkegaard et al., 2015). These findings suggest a potential dissociation where structural enlargement or increased network centrality might coexist with, or perhaps represent an attempt to compensate for, reduced regional functional activation.

Intriguingly, the relationship between network properties and treatment outcomes offers another perspective. In Meige syndrome, patients with better STN (subthalamic nucleus)-DBS (deep brain stimulation) outcomes showed higher preoperative nodal centrality (betweenness/degree) and enhanced connectivity in the left precentral gyrus and bilateral postcentral gyri compared to poor responders (Liu et al., 2024). Furthermore, preoperative GMV in the left precentral gyrus and left thalamus positively correlated with symptomatic improvement rates (Liu et al., 2024). This suggests that a structurally “stronger” or more central sensorimotor node preoperatively might indicate a greater capacity for beneficial network reorganization post-intervention. This argues against hyper-centrality being purely maladaptive; instead, it might reflect a network state that, while abnormal, is more amenable to therapeutic modulation or is simply less degraded in individuals who respond better to treatment. Therefore, the observed sensorimotor and insular hyper-centrality likely reflects a complex interplay. It could be partially compensatory for underlying functional deficits or inefficient processing elsewhere in the network. Yet, its association with better DBS outcomes in related conditions suggests it might also signify a preserved capacity for adaptive plasticity, rather than being solely a maladaptive driver of symptoms. Distinguishing these possibilities definitively will require longitudinal studies integrating multimodal imaging across different dystonia subtypes and treatment responses (Liu et al., 2024). Distinguishing these possibilities requires further investigation integrating longitudinal data, different dystonia subtypes, and multiple imaging modalities (Zhu et al., 2023).

### Complex connectivity reorganization across large-scale networks

The NBS analysis revealed a complex subnetwork of altered FC within and between large-scale brain networks (SMN, LSN, FPN, DMN), characterized by both abnormally strong and weak connections. This finding is highly consistent with the broader literature on BSP and other focal dystonias, which increasingly points towards widespread, bidirectional connectivity changes rather than simple hypo- or hyper-connectivity within isolated circuits (Giannì et al., 2022; Huang et al., 2017; Huang et al., 2023; Hanekamp and Simonyan, 2020; Battistella et al., 2016; Battistella and Simonyan, 2019; Battistella et al., 2015; Cheng et al., 2023; Chirumamilla et al., 2019; Delnooz et al., 2013; Fang et al., 2021; Fuertinger and Simonyan, 2017; Marapin et al., 2023). This complex pattern likely reflects the interplay between primary pathological processes and secondary network-wide consequences, including compensatory mechanisms and maladaptive plasticity (Zhu et al., 2023; Evinger, 2015; Mascia et al., 2020; Vo et al., 2023). This FC reorganization affects not only the classic motor control circuits (cortico-striato-thalamo-cortical circuit) but also profoundly impacts the networks like the FPN and DMN. The FPN is crucial for executive functions, attention, and goal-directed behavior (cognitive control) (Huang et al., 2017; Zanto and Gazzaley, 2013), while the DMN is primarily involved in self-referential thought, mind-wandering, and episodic memory retrieval (Smallwood et al., 2021; Menon, 2023). Abnormalities in these networks and their interactions with SMN highlight that the pathophysiological impact profoundly affects these higher-order cognitive and self-referential systems, which may significantly contribute to the non-motor symptoms frequently accompanying blepharospasm (Ferrazzano et al., 2019; Scorr et al., 2022; Huang et al., 2017; Yang et al., 2016; Hou et al., 2022). Therefore, understanding blepharospasm as a network disorder characterized by complex reorganization of large-scale brain connectivity provides a crucial theoretical foundation for future exploration of more precise diagnostic biomarkers and network-based therapeutic strategies, such as neuromodulation (Mascia et al., 2020; Huang et al., 2023).

### Strengths, clinical implications, and future directions

A key strength of this study is the focus on untreated BSP patients, minimizing the confounding neuroplastic effects induced by treatments like BoNT (Jochim et al., 2018; Feng et al., 2021; Brodoehl et al., 2019), thereby providing a clearer window into the intrinsic network state associated with the disease. Our findings underscore BSP as a disorder of distributed neural networks. The identified metrics, particularly the nodal efficiency of the left thalamus (correlating with duration) and right precentral gyrus (correlating with severity), show promise as potential imaging biomarkers for tracking disease progression or severity, although validation in larger, independent cohorts is crucial. The dysfunctional nodes (thalamus, SMA, precentral gyrus, postcentral gyrus, insula) and the altered connectivity patterns represent potential targets for therapeutic interventions, including targeted neuromodulation strategies.

Limitations include the cross-sectional design, modest sample size common in neuroimaging, and the indirect nature of rs-fMRI. In addition, we used binary networks based on absolute correlation thresholds. While calculating metrics across a range of thresholds and using the AUC mitigates dependency on a single arbitrary choice, this approach discards information about the weight or strength of the connections. Future studies should incorporate weighted network analysis or apply proportional thresholding to ensure consistent network density across participants, which could provide a more nuanced understanding of topological alterations. Another limitation is the reliance on the AAL atlas. The AAL atlas defines regions anatomically, which may not align perfectly with functional boundaries, and its coarse parcellation of subcortical structures and the cerebellum may obscure more subtle, localized network changes. Given the well-established role of the cerebello-thalamo-cortical circuit in dystonia, future validation of our findings using higher-resolution, functionally defined parcellations (e.g., the Schaefer-Yeo atlas) that include detailed cerebellar subregions is essential. Furthermore, the generalizability of our findings may be limited due to the single-center study design and the relatively homogenous demographic characteristics of our cohort. Validating these network biomarkers will require multi-center studies to confirm their robustness and generalizability across diverse patient populations. Future research should also prioritize longitudinal studies to map network changes over time and in response to treatment. Integrating multimodal imaging, combining rs-fMRI graph theory with structural MRI (VBM, cortical thickness), DTI (tractography), and potentially PET, will provide a more holistic understanding. Task-based fMRI, particularly involving symptom provocation (specific eye movements, visual stimuli), could complement resting-state findings. Comparing network topologies across different dystonia subtypes using harmonized methods and exploring genotype-connectome relationships will further refine our understanding.

## Conclusion

In conclusion, using a graph theory analysis of rs-fMRI data in untreated BSP patients, this study identified a distinct pattern of functional network disruption involving reduced global local efficiency, decreased functional importance of key thalamic and SMA hubs, increased functional importance of sensorimotor and insular cortical regions (correlated with severity), and complex, bidirectional connectivity changes across distributed brain networks. These findings reinforce the conceptualization of BSP as a large-scale network disorder rooted in dysfunctional sensorimotor control circuits and associated maladaptive/compensatory neuroplasticity within cortico-basal ganglia-thalamocortical loops. This network perspective offers potential pathways for developing objective biomarkers and guiding novel therapeutic strategies, emphasizing the need for continued multimodal and longitudinal investigations into the complex neurobiology of dystonia. Future studies should explore whether these network metrics can serve as predictive biomarkers for treatment response to BoNT or neuromodulation.

## Data Availability

The raw data supporting the conclusions of this article will be made available by the authors, without undue reservation.
